# Allelic and Genotypic Distribution of rs1801133 in the Methylene Tetrahydrofolate Reductase (MTHFR) Gene Among Family Trios Affected by Nonsyndromic Cleft Lip and Palate

**DOI:** 10.7759/cureus.106049

**Published:** 2026-03-29

**Authors:** Praveen Kumar M, Kumar Satish Ravi, Srinivas Reddy Gosla, Vasavi Mohan, Praveen Kumar Neela, Nainika Krishnan, Fatema Ali Khan

**Affiliations:** 1 Department of Anatomy, National Institute of Medical Sciences and Research, NIMS University Rajasthan (formerly National Institute of Medical Sciences and Research), Jaipur, IND; 2 Department of Oral and Maxillofacial Surgery, Gosla Srinivas Reddy (GSR) Institute of Craniomaxillofacial and Facial Plastic Surgery, Hyderabad, IND; 3 Department of Genetics and Molecular Medicine, Vasavi Medical and Research Centre, Hyderabad, IND; 4 Department of Orthodontics and Dentofacial Orthopaedics, Kamineni Institute of Dental Sciences, Narketpally, IND

**Keywords:** cleft lip, cleft palate, ethnicity, nonsyndromic, polymorphism

## Abstract

Cleft lips and/or palates are common congenital anomalies with different prevalence across different populations and geographic regions. The present study analyzed the allelic and genotypic frequencies of the MTHFR rs1801133 polymorphism in familial cleft lip and/or palate trios. This retrospective genetic family-based study was carried out in a population-specific background using samples collected from the data bank of 4,000 patient records from a high-volume craniofacial hospital. Forty familial case-parent trios (n = 120 subjects, 40 unaffected fathers, 40 unaffected mothers, and 40 affected children) were selected. Genomic DNA was extracted from peripheral blood samples, and genotyping of the MTHFR rs1801133 polymorphism was carried out using the polymerase chain reaction-restriction fragment length polymorphism method. Allelic and genotypic frequencies were calculated using IBM Corp. Released 2020. IBM SPSS Statistics for Windows, Version 26. Armonk, NY: IBM Corp., with descriptive statistical analyses applied. Hardy-Weinberg equilibrium and the transmission disequilibrium test were performed using PLINK 1.9 (Purcell Lab, Harvard Medical School). The CC genotype was found to be the most common in all three groups (72.5% of fathers, 85.0% of mothers, and 90.0% of affected children), according to the genotype distribution of the MTHFR rs1801133 polymorphism among the 40 case-parent trios. The CT genotype was observed in 27.5% of fathers, 15.0% of mothers, and 10.0% of affected children. The C allele was observed more commonly in mothers (92.5%), fathers (86.25%), and affected children (95.0%). The T allele was observed in 13.75% of fathers, 7.5% of mothers, and 5.0% of affected children. Transmission disequilibrium test analysis showed no statistically significant preferential transmission of the T allele (OR = 0.4, p = 0.1088), indicating no association. This study contributes to global data on ethnic variation, helping researchers compare populations and design more accurate, population-specific genetic studies.

## Introduction

Cleft lip and/or palate (CL/P) is one of the most common congenital craniofacial anomalies, with a global prevalence estimated to be approximately 1 in 700-1000 live births, although this varies widely across different populations and geographic regions. Recent studies continue to report similar prevalence ranges, with slightly higher or lower frequencies depending on ethnicity and environmental factors. In India, the incidence is reported to range from approximately 1:800 to 1:1000 live births, highlighting the significant burden of this condition in the population [1]. Among the candidate genes implicated in the pathogenesis of nsCL/P, the methylenetetrahydrofolate reductase (MTHFR) gene has received considerable attention due to its critical role in folate metabolism. The MTHFR enzyme catalyzes the conversion of 5,10-methylenetetrahydrofolate to 5-methyltetrahydrofolate, which is a key step in the remethylation of homocysteine to methionine. This reaction is essential for DNA synthesis, repair, and methylation, processes that are particularly important during craniofacial development. Family-based studies have indicated that genetic factors play a significant role in the etiology of nsCL/P [2].

Cleft lip and palate are developmental anomalies that arise during early embryogenesis due to the failure of fusion of facial prominences. Craniofacial development is largely derived from neural crest cells that migrate into the first pharyngeal arch, forming the maxillary and mandibular prominences. The upper lip develops through fusion of the medial nasal and maxillary prominences between the 5th and 7th weeks of gestation. The palate forms from palatal shelves arising from the maxillary prominences between the 6th and 12th weeks, which initially grow vertically, elevate to a horizontal position, and fuse in the midline. Disruption of neural crest cell migration, growth, elevation, or fusion of these structures can result in cleft lip and/or palate [3].

The rs1801133 (C677T) single-nucleotide polymorphism (SNP) in the MTHFR gene results in an alanine-to-valine substitution, leading to the formation of a thermolabile variant of the enzyme with reduced activity. Individuals carrying the TT genotype exhibit significantly lower enzymatic function, resulting in elevated homocysteine levels and impaired folate metabolism. Studies have investigated the potential association between the MTHFR C677T polymorphism and nsCL/P risk [4], with inconsistent results across populations. Some studies reported no association, and subgroup analyses by ethnicity/case source further supported this lack of association [5].

This study aimed to determine the allelic and genotypic distribution and evaluate potential familial transmission patterns of the MTHFR rs1801133 polymorphism among case-parent trios with nonsyndromic cleft lip and/or palate in the Telangana population.

## Materials and methods

Approval for this genetic study was attained from the Institutional Ethics Committees of NIMS University Rajasthan, Jaipur (IEC/P-505/2024) and GSR Institute of Craniofacial and Plastic Surgery (GSRICES), Hyderabad (No/GSR ICFS/2024/001-N). The study adhered to the ethical principles established by the Declaration of Helsinki.

A total of 4,000 patient records were reviewed from the GSR Institute of Craniofacial Surgery (GSRICES), Hyderabad, spanning four years from September 2020 to August 2024. These patients had been treated for cleft lip and/or palate (CL/P), and the objective was to identify familial cases of nsCL/P. Demographically, participants were residents of Telangana for at least five generations.

From the total of 4,000 patient records, familial cases were identified through systematic screening based on documented family history and clinical diagnosis. Only those cases fulfilling predefined inclusion criteria, including confirmed nonsyndromic status and availability of both biological parents, were included. This resulted in the selection of 40 eligible case-parent trios.

Familial aggregation was verified using documented family history obtained from clinical records. A purposive sampling approach was adopted, and all eligible familial cases identified during the study period were included to minimize selection bias.

Each of these families included one affected child and both unaffected biological parents, constituting 40 complete case-parent trios (totaling 120 individuals). The mean age of affected individuals was 6.95 years (approximately 6 years and 11 months), with ages ranging from infancy to adulthood. Among the affected individuals, 23 (57.5%) were males, and 17 (42.5%) were females. This case-parent trio design inherently controls for population stratification and familial genetic background. 

The participants were selected based on the following criteria.

Inclusion criteria

The participants were required to have a (1) confirmed diagnosis of nonsyndromic cleft lip and/or palate in the child, (2) availability of both biological parents, (3) verified positive family history of clefting, and (4) to be residents of Telangana.

Exclusion criteria

The participants with (1) incomplete or unreliable family history and (2) non-biological parents or adoptive parents were excluded.

All participants provided written informed consent before being enrolled in the study. Confidentiality and data privacy were rigorously maintained throughout the study, and all procedures adhered to institutional and Helsinki guidelines.

**Figure 1:**
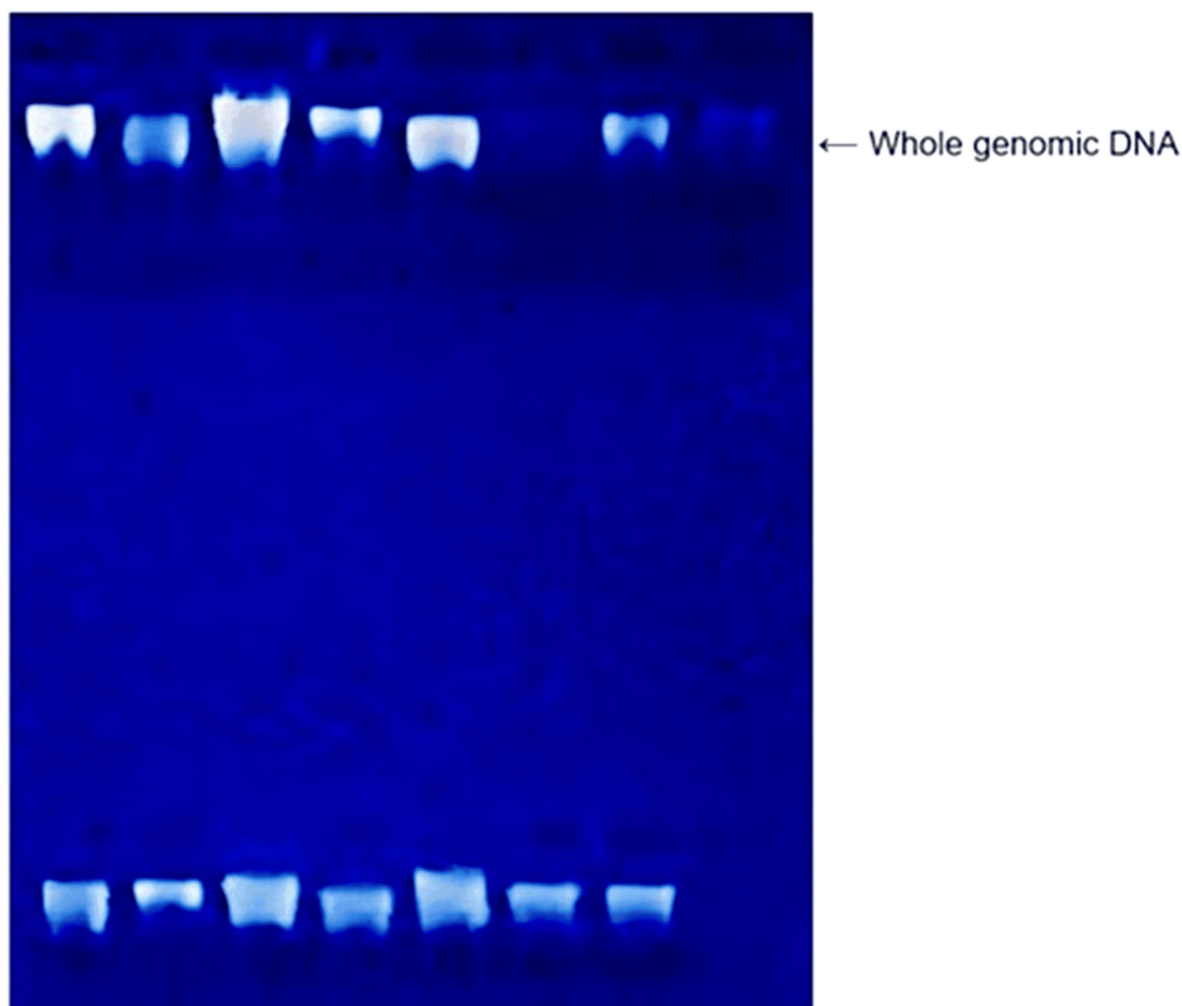
Displays the genomic DNA isolated from the samples.

Venous blood (3 mL) was collected from the median cubital vein in the cubital fossa under aseptic conditions from all participants into ethylenediaminetetraacetic acid (EDTA) vacutainers, which were transported in gel packs to maintain a temperature of 2 to 8°C until delivery to the Department of Genetics, Vasavi Medical and Research Centre. DNA was extracted using the salting-out method, and the quality of the genomic DNA was measured using 0.8% agarose gel electrophoresis (Figure 1). Standard laboratory reagents were used for DNA extraction and PCR procedures, including QIAamp DNA Mini Kit (Qiagen, USA), Taq DNA polymerase, dNTPs, MgCl₂, agarose, ethidium bromide, and restriction enzymes for PCR-RFLP analysis [6,7].

**Figure 2:**
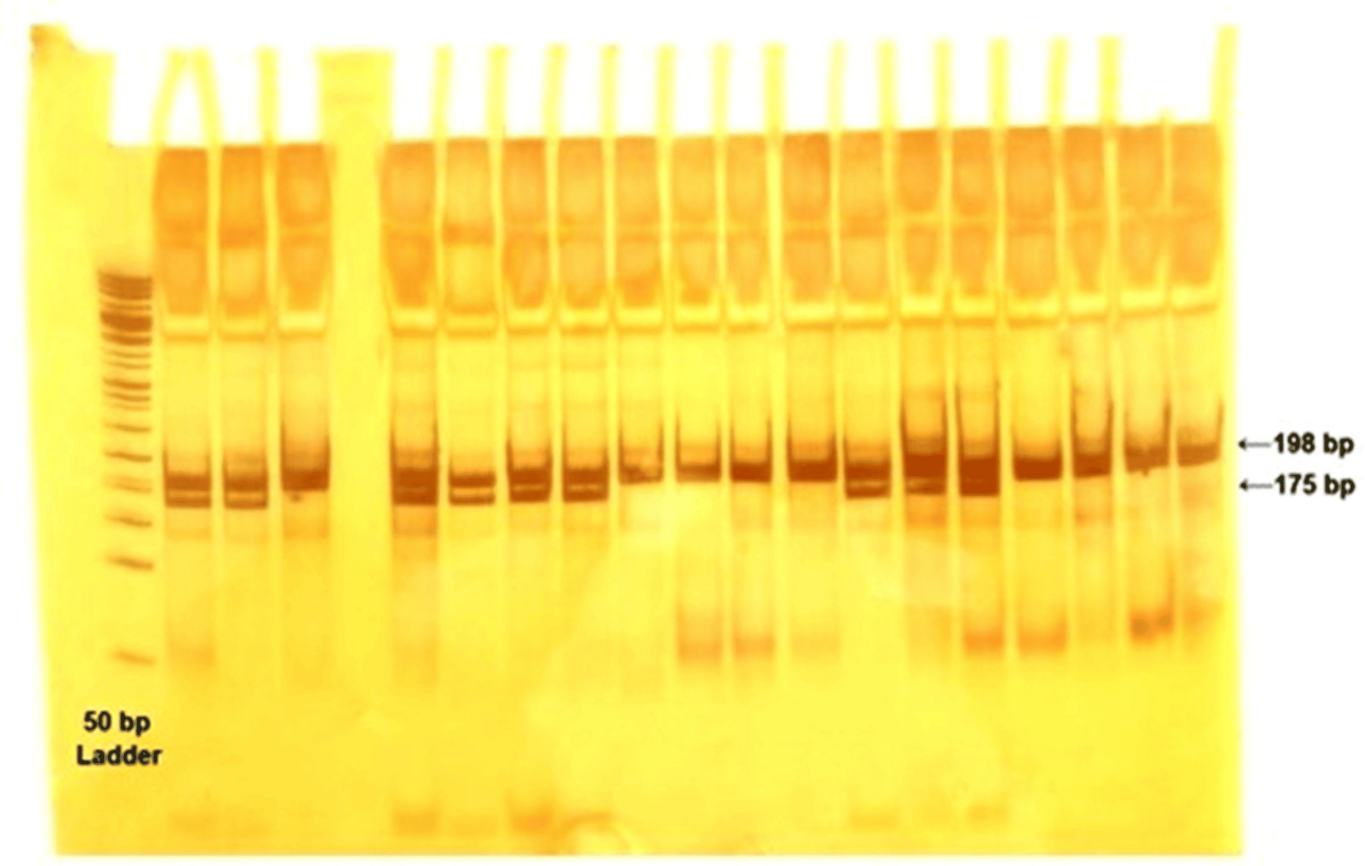
PCR amplification of the MTHFR rs1801133 gene using a 50 bp ladder, producing 198 bp and 175 bp fragments visible on the gel.

Amplification of the MTHFR gene fragment containing the rs1801133 (C677T) polymorphism was carried out by polymerase chain reaction (PCR) using the following primers: forward primer 5′-TGAAGGAGAAGGTGTCTGCGGGA-3′ and reverse primer 5′-AGGACGGTCCGGTGAGAGTG-3′ [8] with the product length of 198 bp and 175 bp. The PCR mix (25 μL total volume) included TaqMan master mix components: water (17.75 μL), buffer (2.5 μL), dNTPs (0.5 μL), MgCl₂ (2 μL), forward primer (0.5 μL), reverse primer (0.5 μL), Taq polymerase (0.25 μL), and DNA template (1 μL). The amplification conditions included an initial denaturation at 95°C for 5 minutes, followed by 35 cycles of 95°C for 45 seconds, 60°C for 30 seconds, and 72°C for 1 minute, with a final extension at 72°C for 10 minutes. Using a gel documentation system, the PCR product was analyzed using 2% agarose gel electrophoresis and visualized under ultraviolet light. The amplified product was further subjected to polymerase chain reaction-restriction fragment length polymorphism (PCR-RFLP) analysis using 10% polyacrylamide gel electrophoresis (PAGE) followed by separation of fragments as shown in Figure 2. (198 bp for CC, 75 bp for TT, and both for CT) [9]. The genotype data obtained from the RFLP method were tabulated and subjected to statistical evaluation.

Standardized laboratory protocols were followed throughout the study to ensure consistency and minimize genotyping errors.

Sample size calculation

The sample size was estimated using a standard epidemiological formula based on the prevalence of cleft lip and palate, which occurs globally at a frequency of approximately 1 in 500-1000 live births and in India at an incidence of approximately 1.3-1.7 per 1000 births. This calculation yielded a sample size of approximately 40 trios. However, given that nearly 70% of cases are nonsyndromic and only 1-2% represent familial cases, the study population is relatively rare. Therefore, the final sample size of 40 case-parent trios reflects both the calculated estimate and the practical feasibility of recruiting eligible familial cases within the study period.

Statistical analysis

Statistical analysis was performed using IBM Corp. Released 2020. IBM SPSS Statistics for Windows, Version 26. Armonk, NY: IBM Corp. and PLINK software version 1.9. Allele and genotype frequencies were calculated from observed genotype counts. Hardy-Weinberg equilibrium (HWE) was assessed in the parental population. The transmission disequilibrium test (TDT) was performed using PLINK v1.9 to evaluate preferential allele transmission within the case-parent trio design, where transmitted alleles were compared with non-transmitted parental alleles. A p-value <0.05 was considered statistically significant.

## Results

Genotyping was performed on 40 case-parent trios, which included 40 affected children and their biological parents (40 fathers and 40 mothers). The genotype distribution of the MTHFR gene rs1801133 polymorphism in the 40 case-parent trios is shown in Table 1.

**Table 1:** Genotype distribution of MTHFR rs1801133 polymorphism among case-parent trios. Data is presented as numbers (n) and percentages (%).

Genotype	Fathers (n = 40)	Mothers (n = 40)	Affected Children (n = 40)
CC	29 (72.5%)	34 (85.0%)	36 (90.0%)
CT	11 (27.5%)	6 (15.0%)	4 (10.0%)
TT	0 (0%)	0 (0%)	0 (0%)

The genotype distribution revealed that the CC genotype was the most common in all groups, with 29/40 (72.5%) fathers, 34/40 (85.0%) mothers, and 36/40 (90.0%) affected individuals having the CC genotype. The CT genotype was found in 11/40 (27.5%) fathers, 6/40 (15.0%) mothers, and 4/40 (10.0%) affected individuals, while the TT genotype was not found in any of the groups. 

Table 2 shows the allelic distribution of the MTHFR gene rs1801133 polymorphism.

**Table 2:** Allele distribution of MTHFR rs1801133 polymorphism among case-parent trios. Data is presented as numbers (n) and percentages (%).

Allele	Alleles in Fathers (n = 80)	Alleles in Mothers (n = 80)	Alleles in Affected Children (n = 80)
C	69 (86.25%)	74 (92.5%)	76 (95.0%)
T	11 (13.75%)	6 (7.5%)	4 (5.0%)

To assess genetic equilibrium and allele transmission patterns, HWE and TDT analyses were performed.

HWE analysis of parental genotypes (Table 3) showed no significant deviation (p = 0.589), indicating that the genotype distribution was showing no deviation from Hardy-Weinberg equilibrium and supporting the reliability of genotyping.

**Table 3:** Hardy–Weinberg equilibrium (HWE) analysis of parental genotypes for the MTHFR rs1801133 polymorphism. A1: Minor allele; A2: Major allele.

CHR	SNP	A1	A2	GENO	O(HET)	E(HET)	p-value
1	rs1801133	T	C	0/17/63	0.2125	0.1899	0.589

TDT analysis (Table 4) revealed that the T allele was transmitted 4 times and untransmitted 10 times from heterozygous parents (OR = 0.4, χ² = 2.571, p = 0.1088), indicating no statistically significant preferential transmission. 

**Table 4:** Transmission Disequilibrium Test (TDT) results for MTHFR rs1801133 polymorphism. T: Transmitted allele count; UT: Untransmitted allele count. OR: Odds ratio.

CHR	SNP	BP	A1	A2	T	UT	OR	CHISQ	p-value
1	rs1801133	11796321	T	C	4	10	0.4	2.571	0.1088

The C allele was the most abundant, with 69/80 (86.25%) fathers, 74/80 (92.5%) mothers, and 76/80 (95.0%) affected individuals having the C allele, while the T allele was found in 11/80 (13.75%) fathers, 6/80 (7.5%) mothers, and 4/80 (5.0%) affected individuals. 

The above results suggest that the C allele and CC genotype are highly prevalent in this population, with no TT homozygotes present.

## Discussion

The majority of orofacial clefts are nonsyndromic (nsCL/P), with syndromic forms accounting for a smaller proportion [10]. The MTHFR gene plays an important role in encoding a key enzyme involved in folate metabolism, crucial for DNA synthesis, repair, and methylation. This enzyme converts 5,10-methylenetetrahydrofolate into 5-methyltetrahydrofolate, the primary methyl donor in one-carbon metabolism [11].

The MTHFR C677T polymorphism is associated with reduced MTHFR enzyme activity, elevated plasma homocysteine concentrations, and diminished plasma folate levels, which have been hypothesized to increase susceptibility to nsCL/P in some populations [5]. Impairments in these crucial steps during embryogenesis are therefore plausible mechanisms for craniofacial malformations like CL/P [12]. Arezoo Jahanbin and Shotelersuk reported that maternal 677CT or TT genotypes are associated with an increased risk of nsCL/P [13,14]. These observations raise the possibility that, in individuals carrying folate metabolism-related genetic variants such as MTHFR rs1801133, normal folic acid intake may be insufficient to prevent CL/P, highlighting the need for genotype-specific nutritional recommendations and further investigation into gene-environment interactions. 

The rs1801133 polymorphism (C677T) results in three genotypes: CC (wild-type), CT (heterozygous), and TT (homozygous variant). The TT genotype shows the lowest enzymatic activity and is associated with impaired folate metabolism [15]. In our study, the CC genotype was predominant among fathers (72.5%), mothers (85.0%), and affected children (90%). In contrast, a study conducted in the Turkish population reported less CC frequency in fathers (55%), mothers (30%), and affected children (45%) [16]. 

Our study found CT frequencies in fathers (27.5%), mothers (15%), and affected children (10%). In contrast, Nasroen et al. reported higher CT frequencies in the Turkish population, particularly in fathers (50%), mothers (50%), and affected children (43%) [16]. The findings of a meta-analysis conducted by Jugessur et al. (2009) found that individuals with the TT genotype had a 1.5-fold increased risk of nsCL/P compared to those with the CC genotype [17]. 

A study conducted by Nasroen et al. in the Turkish population reported TT percentages as fathers (2%), mothers (20%), and affected children (5%) [16]. The homozygous TT genotype was prevalent in Northern China (20%), Southern Italy (26%), and Mexico (32%) [18]. However, the TT allele was found to be absent in our study. 

In our familial case-parent trio analysis, the allelic distribution of the MTHFR rs1801133 polymorphism demonstrated a clear predominance of the C allele across all family members. The C allele frequency was 86.25% in fathers, 92.5% in mothers, and 95.0% in affected children. In contrast, the T allele occurred at relatively low frequencies, observed in 13.75% of fathers, 7.5% of mothers, and 5% of affected offspring. The high prevalence of the C allele and the predominance of the CC genotype, together with the absence of the TT genotype among all trios, suggest that the rs1801133 T allele may have only a limited contribution to the familial transmission of nsCL/P in this population. These findings are in agreement with previous reports from several Asian populations, including studies from India, where the T allele has also been reported to occur at comparatively low frequencies [11]. 

Interestingly, although our results do not support a major role for the T allele in familial CL/P inheritance in this population, other studies within the Indian subcontinent have shown slightly different patterns. For example, Jyotsna et al. reported a significant association between the T allele and increased susceptibility to nsCL/P in South India [15], while Juhi Yadav et al. observed a higher T allele frequency in nsCL/P cases from North India [19]. This suggests that geographic and ethnic variation within India may influence the genetic risk associated with the MTHFR gene. 

Moreover, Wilcken et al. documented a notably higher prevalence of the T allele in Brazilian populations [18], where a stronger association with orofacial clefts has been reported, reinforcing the notion of ethnic and geographic heterogeneity in MTHFR allele distribution and its potential role in craniofacial anomalies. 

The familial context of our study strengthens the conclusion that the C allele is more frequently transmitted from parents to affected children in this cohort, and the lack of T allele homozygosity (TT genotype) in all trios further diminishes the likelihood of its involvement in familial nsCL/P pathogenesis in this population. This aligns with a study conducted by Pamela Kelly Farias de Aguiar, which revealed no preferential transmission of the T alleles in the Brazilian population [11].

Collectively, these results indicate that the MTHFR rs1801133 polymorphism may have varying roles across different ethnic groups [18]. Further research, including a larger sample size and additional functional variants involved in folate metabolism, is warranted to better understand the complex genetic architecture of cleft lip and palate.

Our findings are consistent with the meta-analysis by Alghonemy and Ashmawy, who observed that the MTHFR c.677C>T polymorphism is associated with an increased risk of NSCL/P in certain populations, though it does not show a significant effect when all groups are pooled together, with the CC and TT genotypes more common in controls and the CT genotype more frequent in cases [20], highlighting the relevance of folate metabolism genes in the development of these defects. Taken together, these data support the idea that MTHFR C677T influences NSCL/P risk in a way that depends on the genetic background and population being studied [5,20]. 

The present study did not demonstrate a statistically significant association between the MTHFR rs1801133 polymorphism and nonsyndromic CL/P based on TDT analysis. This finding is consistent with the lack of preferential allele transmission observed in the present study. The observed trend may be influenced by a limited sample size. 

The absence of the TT genotype may be attributed to the relatively low frequency of the T allele and limited sample size, rather than genotyping error, as supported by the Hardy-Weinberg equilibrium.

Most previous studies have focused on non-familial case-control populations, whereas the present study used a family-based trio design. Therefore, direct comparisons should be made with caution; however, similar trends in allele distribution can still be observed.

Future directions

Future research should explore how rs1801133 behaves across different populations, as its role may vary depending on genetic background. Conducting large-scale genome-wide association studies (GWAS) in diverse groups can help identify other genetic factors that may work alongside this variant.

It would also be valuable to examine how genetic variation interacts with environmental influences such as dietary folate intake and maternal lifestyle. A better understanding of these combined effects may provide deeper insight into the biological mechanisms involved in CL/P and support more informed approaches to its management.

Limitations

This study has several limitations, including a relatively small sample size, a lack of genetic power calculation, the absence of genotype validation using Sanger sequencing, and a focus on a single SNP. These limitations may affect the generalizability of the findings; therefore, the results should be interpreted with caution and require validation in larger cohorts.

## Conclusions

This study examined the allelic and genotypic distribution of the MTHFR rs1801133 polymorphism in family trios affected by nonsyndromic cleft lip and palate. The results showed that the CC genotype was the most common among both affected children and their parents, while the TT genotype was not observed in any participants. TDT analysis demonstrated no statistically significant preferential allele transmission (OR = 0.4, p = 0.1088). Overall, no clear association was observed between the MTHFR rs1801133 polymorphism and nonsyndromic CL/P in this study population. The absence of the TT genotype may be attributed to the low frequency of the T allele and the limited sample size. These results differ from some previous studies conducted in other regions, highlighting possible population-specific variations. Further research with larger sample sizes and additional genetic factors is needed to better understand these differences.
